# Wind conditions on migration influence the annual survival of a neotropical migrant, the western yellow-breasted chat (*Icteria virens auricollis*)

**DOI:** 10.1186/s12898-017-0139-7

**Published:** 2017-08-10

**Authors:** Andrew C. Huang, Christine A. Bishop, René McKibbin, Anna Drake, David J. Green

**Affiliations:** 1 Environment and Climate Change Canada, Delta, BC Canada; 2 Department of Biological Sciences, Center for Wildlife Ecology, Simon Fraser University, Burnaby, BC Canada; 3 Department of Forest and Conservation Sciences, University of British Columbia, Vancouver, BC Canada

**Keywords:** Climate conditions, Wind, Storm, Yellow-breasted chats, Neotropical migrants, Climate change

## Abstract

**Background:**

Long-distance migratory birds in North America have undergone precipitous declines over the past half-century. Although the trend is clear, for many migrating species underpinning the exact causes poses a challenge to conservation due to the numerous stressors that they encounter. Climate conditions during all phases of their annual cycle can have important consequences for their survival. Here, using 15 years of capture-recapture dataset, we determined the effects of various climate factors during the breeding, wintering, and migrating stages on the annual survival of a western yellow-breasted chat (*Icteria virens auricollis*) population breeding in southwestern Canada.

**Results:**

El Niño effects over the entire annual cycle had little influence on the annual apparent survival of yellow-breasted chats. However, we found evidence that wind conditions during migration, specifically average westerly wind speed or the frequency of storm events, had significant adverse effects on adult annual apparent survival. In comparison, precipitation levels on wintering ground had little to no influence on adult annual apparent survival, whereas growing degree days on the breeding ground had moderate but positive effects.

**Conclusions:**

In the face of climate change and its predicted impacts on climate processes, understanding the influence of weather conditions on the survival of migrating birds can allow appropriate conservation strategies to be adopted for chats and other declining neotropical migrants.

**Electronic supplementary material:**

The online version of this article (doi:10.1186/s12898-017-0139-7) contains supplementary material, which is available to authorized users.

## Background

Widespread declines in bird populations are evident across much of North America, with recent findings revealing that an alarming one-third (37%) of North American bird species are of high conservation concern [1]. Long-distance neotropical migrants have declined more steeply than residents and short-distance migrants [1, 2]. Longer journeys mean that these birds encounter more potential impediments along their migratory routes, including collisions with man-made infrastructures, light pollution, increased predation risk, and inadequate food sources [3, 4]. In addition, anthropogenic and environmental threats on the breeding and wintering grounds add to the suite of stressors for neotropical migrants [5, 6]. Unfavourable weather conditions are one of the most detrimental factors that can compromise their survival and reproductive phenology [7–11]. Having a more holistic understanding of the climatic processes and their impacts on migratory birds would allow conservation strategies to be effectively implemented for these species [12].

Migratory birds have endured arduous and perilous journeys between and within North, Central and South Americas for millennia. However, with climate change projections predicting drastic alterations in climatic conditions, uncertainty exists about the future persistence of migratory bird populations [13]. The predicted increase in the frequency, intensity, and duration of extreme weather events—including droughts, intense precipitation, and windstorms—are expected to have adverse effects on avian population dynamics [8, 10, 14, 15]. Neotropical migrants may be particularly susceptible to extreme climate variability, as climate fluctuations and anomalies can result in direct mortality and/or impact food availability during all stages of their annual cycle: breeding, wintering, and migration [16]. Understanding how climatic conditions at different stages of the annual cycle influence the survival of neotropical migrants is critical in evaluating how climate change will impact migratory birds [17, 18].

The objective of this study was to examine how annual survival of a neotropical migrant, western yellow-breasted chat (*Icteria virens auricollis*; hereafter: chat), is influenced by climatic conditions during its breeding, wintering, and spring migratory periods. We developed capture-mark-recapture models constrained with climate variables to evaluate the effects of: (1) El Niño Southern Oscillation (ENSO) during the entire annual cycle; (2) temperature and precipitation conditions on the breeding grounds; (3) precipitation level on the wintering grounds; and finally (4) wind speed, precipitation level, and number of storm events on the spring migration route. Survival was estimated using 15 years (2001–2015) of capture-recapture data from a breeding population of chats in southern British Columbia, Canada. Our study presents the first comprehensive analysis on how adult survivorship of chats in this endangered population [19] in Canada can be influenced by climate factors throughout three major phases of its annual cycle.

## Methods

### Study species and area

**Fig. 1:**
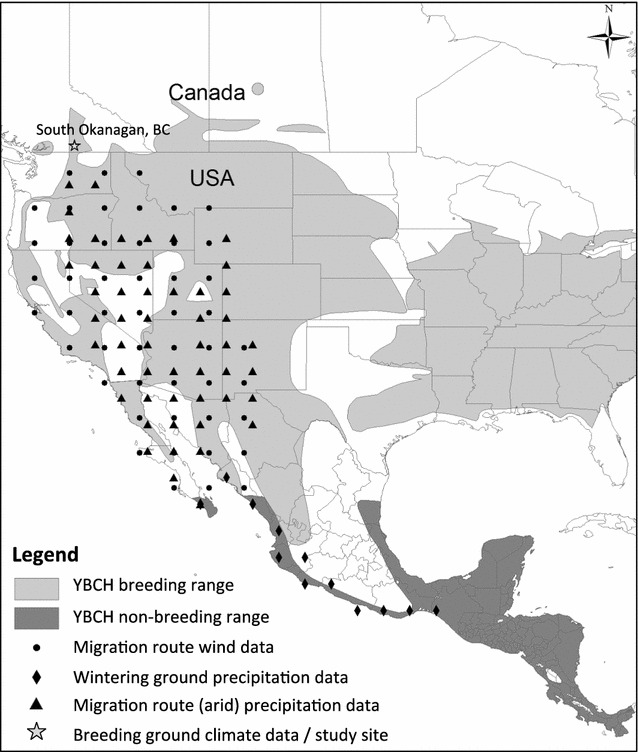
Location points used to collect climate data from the approximated migration route (wind = *circle*; precipitation = *triangle*), wintering ground (*diamond*), and breeding ground/study site (*star*) of yellow-breasted chats (western subspecies). *Shaded* in *light and dark grey* are the breeding and overwintering ranges of yellow-breasted chats (both subspecies), respectively, adapted from IUCN (April 2016)

The yellow-breasted chat is a neotropical migratory songbird with a transcontinental distribution extending from southern Canada, the United States, Mexico, to Central America (Fig. 1). The western *auricollis* subspecies has a fragmented breeding distribution in the west, which includes our study area in the south Okanagan valley of British Columbia, Canada, at the northern tip of the geographic range for this subspecies (Fig. 1). Genetic evidence indicates that the western subspecies overwinters on the westcoast of Mexico (southern Baja California, Sinaloa to Oxaca) [20]. Chats in the south Okanagan valley nest in low elevation (>500 m) riparian thickets dominated by wild rose (*Rosa* spp.), snowberry, and other native shrub species [21]. Their diet consists primarily of insects, but also includes fruits and rose petals [22, 23]. We colour-banded, monitored, and resighted birds breeding in the Okanagan valley from Penticton (49º 27′ N, 119º 36′ W) to Osoyoos (49º 1′ N, 119º 26′ W) on the USA border, a distance of 66 km between 2001 and 2015. To ensure that resighted individuals were accurately determined, colour combos were confirmed upon multiple observations and by at least two observers (see McKibbin and Bishop [23, 44] for details). Birds were sexed and aged (second-year [SY] or after second-year [ASY]) based on plumage characteristics and molt limits [24]).

### Climate data and models

#### El Niño Southern Oscillation (ENSO)

ENSO is known to influence a broad spectrum of climate factors in North America including precipitation anomalies in Mexico and southern California [25–27], temperature fluctuations [28], and wind conditions [7, 29]. Climate indices describing ENSO conditions (e.g. El Niño-Southern Oscillation Precipitation Index [ESPI], Southern Oscillation Index [SOI]) have been found to influence the survival rate of several neotropical migrants (e.g. Swainson’s Thrush *Catharus ustulatus* [9]; Black-throated Blue Warblers *Setophaga caerulescenes* [18]; Yellow Warblers *Setophaga petechia* [30, 31]). We examined the combined effects of ENSO on the survival rate of chats using the average SOI from May to April (“Model set 1”; Table 1). SOI is a standard index that measures large-scale fluctuations in air pressure occurring between the western and eastern tropical Pacific, and provides a robust index for tracking ENSO phases. We obtained standardized monthly SOI values from the Climate Prediction Center: http://www.cpc.ncep.noaa.gov/data/indices/soi.

**Table 1:** Annual and region-specific climate data used to examine climate effects on annual apparent survival of western yellow-breasted chats breeding in the south Okanagan valley, British Columbia, Canada

Model set #	Life cycle stage	Climate variable	Calculated by
1	Annual	Southern Oscillation Index (SOI_MAY–APR_)	Average monthly standardized Southern Oscillation Index (SOI)
2	Breeding ground	Growing degree days (GDD_JAN–MAY_)	Sum positive GDD, where GDD = ([T_MAX_ + T_MIN_]/2)—10
		Precipitation_OCT–APR_ Precipitation_MAY–JUL_	Sum daily rainfall (mm)
3	Wintering ground	Precipitation_MAY–NOV_ Precipitation_DEC–APR_	Sum daily rainfall (mm)
4	Migration route	Precipitation_NOV–MAY(ARID)_	Sum daily rainfall (mm)
		Precipitation_NOV–MAY(DESERT)_	Sum daily rainfall (mm)
		Westerly wind speed (U-wind_APR–MAY_)	Average mean daily U-wind speed (m/s) from 6 p.m.–6 a.m.^b^ at 850 and 925 mb^a^
		Southerly wind speed (V-wind_APR–MAY_)	Average mean daily U-wind speed (m/s) from 6 pm – 6am^2^ at 850mb and 925mb^a^
		Number of storm nights (Storm_APR–MAY_)	Sum of days with extreme (>95 percentile) U or V wind speeds

^a^Within the altitudinal range of migrant songbirds [67, 68]

^b^Includes 6 p.m., 12 and 6 a.m. (12 pm was excluded) as most migrants begin migration after dusk, peak at midnight, and end before dawn

#### Climate conditions on breeding ground

Precipitation and temperature in the Pacific northwest are influenced by ENSO. When ENSO is in its strong phase (i.e. El Niño), jet stream becomes diverted into California, resulting in low precipitation and increased frequency of summer droughts in the Pacific Northwest [6, 32]. Growing degree days (GDD), a measure of heat accumulation, and precipitation level both influence primary productivity and insect emergence [33–36], and in turn food availability for animals in subsequent trophic levels [37–39]. We therefore predicted that precipitation in two periods (October–April and May–July) and GDD prior to start of breeding (January–May) would have positive effects on the survival of chats (“Model set 2”; Table 1). Climate variables on the breeding ground of south Okanagan valley were extracted and averaged from two local weather stations: “Penticton A” (WMO ID: 71889; 49^o^27′36″ N, 119^o^36′0″ W; elevation 344.4 m) and “Osoyoos CS” (WMO ID: 71215; 49^o^1′48″ N, 119^o^26′24″ W; elevation 282.9 m).

#### Climate conditions on wintering grounds

ENSO has divergent effects on precipitation in western Mexico: El Niño is associated with more rainfall during the winter/spring in northwestern Mexico, whereas La Niña is associated with more summer monsoon rainfall in southwestern Mexico [27]. Rainfall is expected to influence biological productivity on the wintering grounds, and as a result affect the overwinter survival and mass gain of migratory birds prior to spring migration [5, 7]. We therefore predicted that precipitation level on the wintering grounds during the monsoon and/or the late winter would influence the survival of chats (“Model set 3”; Table 1). Modeled rainfall data on the wintering grounds was obtained from the National Center of Environmental Prediction (NCEP)/National Center for Atmospheric Research (NCAR) Reanalysis database, as provided by the NOAA-CIRES Climate Diagnostic Center at Boulder, CO, USA, using R package RNCEP [40]. Precipitation data from the wintering ground was extracted from 12 location points (Fig. 1).

#### Climate conditions on spring migration route

Wind conditions and storm events during the migration period are expected to influence flight costs and survival during flight [7, 8]. In addition, habitat quality at stopover sites can also be affected by precipitation that falls in the winter and spring (November–May). Rainfall in arid and/or desert regions within the flyway may have a disproportionate effect on survival, and variation in precipitation in the desert region of the western flyway was associated with variation in the annual survival of Swainson’s Thrush [9]. We predicted that westerly (U) and southerly (V) wind speeds and the number of storm nights on the migratory flyway during the migratory period (April–May) would have negative effects, and that precipitation in either the desert region or arid region of the flyway would have positive effects on the annual survival of chats (“Model set 4”; Table 1). Modeled climate data on the migration route was also obtained from the NCEP/NCAR Reanalysis database using RNCEP [40]. Precipitation data were extracted from “arid” (N = 52) and “desert” (N = 23) zones within the western flyway [41], whereas wind speed data was extracted from the entire flyway (N = 49; Fig. 1).

### Analysis

We estimated apparent annual adult survival from 2001 to 2015 using the Cormack-Jolly-Seber model. We calculated the probability of an adult returning to the study site (ϕ) after controlling for the probability that banded individuals were resighted or recaptured, hereafter described as resighting probability (*ρ*), using program MARK 5.1 [42, 43]. Probability of return (ϕ) reflects both survival and emigration, and our apparent annual survival estimates therefore underestimate annual survival. The global model that allowed adult survival to vary as a function of gender, age and year and the resighting probability to vary as a function of gender and year fit the data well and showed no evidence of overdispersion (median procedure, ĉ < 1).

We first determined the best model structure for the resighting rate, and then modeled survival rates with candidate models containing gender, age, year, and all possible interactions (Table 1). We then developed a series of candidate model sets to examine whether annual apparent survival varied with annual ENSO (Model set 1), breeding ground conditions (Model set 2), winter conditions (Model set 3), or migration conditions (Model set 4). Model set 2 included models with both Precipitation_OCT–APR_, Precipitation_MAY–JUL_, and GDD, the precipitation variables combined or alone, and GDD alone, in addition to the best model in the earlier temporal analysis (n = 6 models). Model set 3 included models with Precipitation_MAY–NOV_ or Precipitation_DEC–APR_ and the best model in the earlier temporal analysis (n = 3 models). Both variables were not included because they were highly correlated. Model set 4 included models with both U + V-wind, U and V-wind alone, the number of storm nights in April and May, Precipitation_NOV–MAY_ on either the desert or arid region of the migratory flyway, and the best model from the earlier temporal analysis (n = 7 models). Finally, we asked whether overall ENSO conditions or conditions on the breeding grounds, wintering grounds or on migration best described variation in annual apparent survival. In this candidate set we included the top model in each of Model sets 1–4 and the best model in the earlier temporal analysis. We used a hierarchical modelling approach so that we can evaluate climate effects that operate in a single season allowing comparison with other studies focused on one season alone, and then compete the best models from within each season to assess which period plays the most important role in explaining variation in annual survival. With the exception of the annual SOI, all climate variables were standardized by subtracting the mean and dividing the standard deviation allowing estimated effect sizes to be compared directly. At each stage of the analysis we used Akaike’s Information Criterion to rank competing models. Model weights and beta estimates of effect sizes were used to compare models and assess the importance of individual climate variables.

## Results

We colour-banded a total of 313 chats (118 females, 195 males) between 2001 and 2015. Ninety-four birds were resighted or recaptured at least once a year or more after banding with one male resighted for 6 consecutive years after being banded as an SY bird in 2005. Out of the 148 re-encounters, 93 (63%) were resighted but not recaptured; the remaining 55 (37%) were predominantly both recaptured and resighted, with a few individuals being recaptured but not resighted.

### Apparent survival rate

The best resighting model indicated that resighting varied with gender (Table 2); males were more likely to be resighted on the breeding grounds than females (male = 0.59 ± 0.05; female = 0.39 ± 0.09). This model received marginally more support than a model where resighting of males and females did not differ and substantially more support than a model where resighting varied with both gender and year.

**Table 2:** Summary of models examining gender (g), age (a), and temporal (t) variation in (a) resighting probability, and (b) apparent annual survival of the western yellow-breasted chats breeding in south Okanagan valley, British Columbia (n = 313 individuals, 461 encounters

Model	AIC_C_	ΔAIC_C_	Weight	K
(a) Resighting models				
Phi(g*a*t) p(g)	774.28	0	0.51	58
Phi(g*a*t) p(.)	774.30	0.02	0.49	57
Phi(g*a*t) p(g + t)	794.37	20.09	0	71
Phi(g*a*t) p(g*t)	830.29	56.01	0	84
(b) Apparent annual survival models				
Phi(.)	708.67	0	0.24	3
Phi(g)	709.22	0.55	0.18	4
Phi(a)	709.42	0.75	0.17	4
Phi(g + a)	709.47	0.80	0.16	5
Phi(g + a+ga)	709.57	0.90	0.16	6
Phi(g + a+t)	713.22	4.55	0.02	18
Phi(g + a+ga + t)	713.32	4.67	0.02	19
Phi(g + t)	713.97	5.30	0.02	17
Phi(a + t)	714.68	6.01	0.01	17
Phi(t)	714.97	6.30	0.01	16
Phi(g*a*t)	774.28	65.61	0	58

We show all models in the resighting candidate set and all models with ΔAIC_C_ < 10 and the global Phi model in the apparent annual survival candidate set. In all apparent annual survival models resighting probability varies with gender

*Phi(.)* null model, Phi(g*a*t) global model

The best model examining temporal variation in the annual apparent survival of chats suggested that annual apparent survival was relatively constant across the 15 years of the study. The top (i.e. the null model) received slightly more support than models indicating that survival varied with gender and/or age, and substantially more support than models indicating that survival varied across years (Table 2). The estimated annual apparent survival from the top model was 0.57 ± 0.03, similar to the estimate of 0.65 from a previous study [44]. See Additional file 1: Table S1 for estimates of annual survival from the simple temporal model.

### Climate variables predicting survival rates

#### Relationship between climate variables

ENSO conditions over the course of the year, measured using the average SOI from May to April (SOI_MAY–APR_), was postively correlated with average westerly wind speeds and the frequency of storms in April and May (U-wind_APR–MAY_, r = 0.605, p = 0.02; Storm_APR–MAY,_ r = 0.522, p = 0.06). SOI_MAY–APR_ was not significantly correlated with any of the other climate variables (Additional file 1: Table S2). Breeding season climate variables (GDD_JAN–MAY_, Precipitation_OCT–APR_, Precipitation_MAY–JUL_) were not significantly intercorrelated (all r < 0.45, Additional file 1: Table S2). Precipitation on the wintering grounds from May to November was correlated with precipitation on the wintering grounds from December to April (r = 0.816, p = 0.004). Unsurprisingly, average westerly wind speeds on the migration flyway in April and May increased as the frequency of storms increased (r = 0.841, p = 0.002), and precipitation in desert regions of the migration flyway from November to May was highly correlated with precipitation in arid regions of the flyway (r = 0.927, p < 0.001). Across periods the only climate variables that were significantly intercorrelated were precipitation on the breeding grounds from May to July and the frequency of storms on migration (r = 0.615, p = 0.02, Additional file 1: Table S2).

#### El Niño Southern Oscillation (ENSO)

Annual apparent survival of chats was not related to the average ENSO conditions experienced over the course of the year. The model that included the SOI_MAY-APR_ term received less support than the model indicating that annual apparent survival was constant, and the beta estimate for the SOI_MAY–APR_ term had 95% CI intervals that spanned zero (−0.28 ± 0.20, −0.68 to 0.12).

#### Climate conditions on breeding ground

The number of growing degree days was positively associated, and precipitation on the breeding grounds between May and July was negatively associated, with the annual apparent survival of chats. The top model in Model set 2, that examined breeding ground effects on annual survival, included both GDD_JAN–MAY_ and Precipitation_MAY–JUL_. This model received slightly more support than the model with only the GDD_JAN–MAY_ term and 15 times the support of the null model (Table 3). Beta estimates for the GDD_JAN–MAY_ term had 95% CI that did not span zero (0.30 ± 0.13, 0.06–0.55), whereas those for Precipitation_MAY-JUL_ were lower and had 95% CI that spanned zero (−0.23 ± 0.16, −0.53 to 0.08).

**Table 3:** Models examining the relationship between climate variables on the breeding grounds, wintering grounds, and on migration and the apparent annual survival of western yellow-breasted chats breeding in the Okanagan valley, British Columbia, Canada (n = 313 individuals, 461 encounters)

Variables	AIC_C_	ΔAIC_C_	Weight	K
a. Breeding ground				
GDD_JAN–MAY_ + precipitation_MAY–JUL_	704.09	0	0.30	5
GDD_JAN–MAY_	704.34	0.25	0.26	4
GDD_JAN–MAY_ + precipitation_OCT–APR_	705.14	1.05	0.17	5
GDD_JAN–MAY_ + precipitation_OCT–APR_ + precipitation_May–JUL_	705.85	1.76	0.12	6
Precipitation_OCT–APR_	707.78	3.69	0.05	4
Precipitation_MAY–JUL_	708.24	4.15	0.04	4
Precipitation_OCT–APR_ + precipitation_MAY–JUL_	708.52	4.44	0.03	5
Phi(.)	708.67	4.58	0.02	3
Phi(g*a*t)	774.28	70.19	0	58
b. Wintering ground				
Phi(.)	708.67	0	0.44	3
Precipitation_DEC–APR_	709.10	0.42	0.36	4
Precipitation_MAY–NOV_	710.28	1.61	0.20	4
Phi(g*a*t)	774.28	65.61	0	58
c. Migration route				
U-wind_APR–MAY_	702.88	0	0.43	4
Storm_APR–MAY_	703.43	0.55	0.33	4
U-wind_APR-MAY_ + V-wind_APR–MAY_	704.78	1.90	0.17	5
Precipitation_NOV–MAY(ARID)_	708.57	5.69	0.03	4
Phi(.)	708.67	5.79	0.02	3
Precipitation_NOV–MAY(DESERT)_	710.06	7.19	0.01	4
V-wind_APR–MAY_	710.31	7.43	0.01	4
Phi(g*a*t)	774.28	71.4	0	58

*K* number of parameters, *GDD* growing degree days, *Phi(.)* null model, Phi(g*a*t) global model

#### Climate conditions on wintering ground

Precipitation on the wintering grounds was not associated with variation in the annual apparent survival of chats. Models containing the Precipitation_DEC–APR_ and/or the Precipitation_MAY–NOV_ terms received less support than the null model in Model set 3 (Table 3).

#### Climate conditions on spring migration route

**Fig. 2:**
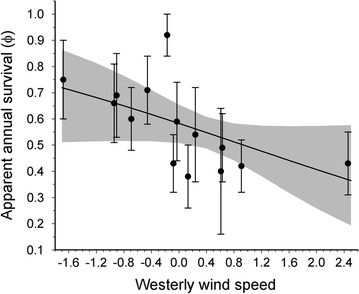
Annual apparent survival (±SE) of adult western yellow-breasted chats in the south Okanagan valley, British Columbia, Canada from 2001 to 2015 in relation to standardized westerly wind speed during migration. *Solid lines* and *shaded area* represent predicted apparent annual survival ±95% CI from the top model

Wind speed on the migration flyway during April and May was negatively associated with the annual apparent survival of chats (Fig. 2). Three models in Model set 4 that examined migration effects on annual survival received strong support, and all three models included terms associated with wind speed and/or frequency of storm events (Table 3). The top model in Model set 4, that included only the westerly wind speed term **(**U-wind_APR–MAY_), received over 20 times the support of the null model. Beta estimates for the U-wind term had confidence intervals that did not span zero (−0.36 ± 0.13, −0.61 to −0.1). The second ranked model that received a similar level of support contained the Storm_APR–MAY_ term, and the third ranked- model included both the U-wind_APR–MAY_ and the V-wind_APR–MAY_ terms. Models with terms associated with precipitation at stopover habitat in desert or arid regions of the migration flyway received negligible support (Table 3).

#### Strongest predicting climate conditions on annual survival

When competing models examining different hypotheses for climate effects on annual apparent survival were tested, the top model indicated that annual apparent annual survival rates were best predicted by westerly wind on the migratory flyway during April and May. This model received nearly twice the support of the model linking annual survival and conditions on the breeding grounds that contained the Precipitation_MAY–JUL_ + GDD_JAN–MAY_ terms (Table 4). See Additional file 1: Table S3 for beta estimates, standard errors and 95% confidence intervals for logit link function parameters in climate and null models in Table 4).

**Table 4:** Comparison of best-supported climate models from the candidate sets examining apparent annual survival of western yellow-breasted chats during each stage of the annual cycle (migration, breeding, wintering), the annual ENSO model and the null model (n = 313 individuals, 461 encounters)

Stage	Model	AIC_C_	ΔAIC_C_	Weight	K
Migration	U-wind_APR–MAY_	702.88	0	0.59	4
Breeding	GDD_JAN–MAY_ + precipitation_MAY–JUL_	704.09	1.21	0.32	5
Annual	Phi(.)	708.68	5.8	0.03	3
Annual	SOI_MAY–APR_	708.86	5.98	0.03	4
Wintering	Precipitation_DEC–APR_	709.097	6.22	0.03	4
Annual	Phi(g*a*t)	774.28	75.4	0	58

*K* number of parameters, *GDD* growing degree days, SOI Southern Oscillation Index, *Phi(.)* null model, Phi(g*a*t) global model

## Discussion

Large-scale climatic phenomenon such as ENSO have profound impacts on regional weather conditions, including temperature, rainfall patterns, and wind conditions. These climate regimes influence the survival and breeding phenology of neotropical migratory birds [7, 9, 31, 45] Temperature and precipitation have major implications on foliage productivity and insect abundance, which in turn can affect the survival of migrating songbirds during all stages of their annual cycle [33–39]. Adverse wind conditions and extreme storm events, in comparison, can either cause direct mortality or result in higher energetic cost for migrating individuals [7, 8]. In this study, we found evidence that the annual adult survival rate of a neotropical migrant, the western yellow-breasted chat, was negatively and most strongly associated with westerly wind speed during the spring migration from the central west coast of Mexico to southwestern Canada. Further, the frequency of storm events in their spring migration route had a negative effect on their annual survival, whereas GDD from January to May on their breeding ground had a positive effect.

The negative effect of wind conditions was either as a result of higher average westerly wind speed or the high frequency of storm events on the migration route of yellow breasted chats. These two climate indices were correlated, and both described variation in annual apparent survival, making it hard to distinguish between the effects of extended periods with high crosswinds and the effects of less frequent extreme events. Previous studies have similarly shown that wind conditions during migration negatively influenced the annual apparent survival of other migratory birds [46], including yellow warblers (*Setophaga petechial* [7]), and chimney swifts (*Chaetura pelagica* [10]). Our 15 years of long-term study corroborated the findings of other shorter term studies that varied from 3 to 9 years (7, 10). Favourable tailwind conditions facilitate migratory flight, and thereby allow birds to expend less energy per unit distance. On the other hand, turbulences and strong winds against the direction of their flight path can result in less efficient migratory flights, leading to greater energy expenditure [47, 48]. Adverse wind conditions deplete their energy reserves, potentially rendering individuals to either die of exhaustion or become more susceptible to depredation risks. Although most migrants stay grounded until windstorms have abated, individuals in the midst of migrating are at most risk of perishing or becoming displaced from these extreme storm events. The effects of windstorms are likely even more detrimental to the survival of migrants when they are flying across long stretches of landmass or waterbodies without suitable stopover sites (9). In the case of chats, the Great Basin, the Sonoran Desert, and the Gulf of Mexico may all act as migratory barriers.

An alternative explanation for the negative association between westerly wind speed and annual apparent survival is that strong westerly crosswinds cause migrating chats to stray off course and breed elsewhere, likely in the eastern or southern parts of their breeding range (7, 49). When faced with prevailing crosswinds, migrants may be pushed off their flight path, or compensate by reorienting their flight to offset the drift in order to remain philopatric to their breeding site at the cost of higher energy expenditure [49–53]. Migrants are more likely to opt for the latter strategy in situations where being blown off course means encountering inhospitable terrains such as oceans and deserts [49]. In our study, chats that experienced strong westerly crosswinds on the pacific flyway could potentially afford to deviate eastward and still encounter potentially suitable stopover or breeding sites. Alternatively, chats may have also settled for more southern breeding grounds when unable to overcome the strong winds or storms, leading to individuals from the Okanagan (northern edge population) to shift further south in years where migratory conditions were unfavourable. This potential population dynamic shift can be confirmed by conducting similar survival estimates in the other populations across its range. Recent technological advances allowing tracking of small birds (e.g. MOTUS towers [54]) may provide an opportunity to assess how chats and other species on different flyways respond to variation in wind conditions during flight [11].

Our study provided evidence that GDD on the breeding ground prior to the breeding season had a positive effect on the annual survival of chats. GDD has strong influences on primary productivity and insect biomass [33, 34], and has been shown to be a key driver of avian distribution and diversity [55, 56]. Plant productivity positively predicts insect abundance [57], which in turn, affects the breeding phenology and success of insectivorous passerines, including dusky flycatchers (*Empidonax oberholseri* [58]), tit species (*Parus* spp. [59]), and horned larks (*Eremophila alpestris* [60]). Further, increased plant growth means denser foliage, potentially allowing for nests to be better concealed from predators and harsh weather [58, 60, 61]. In years where GDD promoted ample food source and possibly better nest concealment in our study area, adult chats may have perceived the breeding habitat as high quality, and as a result demonstrated higher site fidelity.

Abundant rainfall facilitates plant growth and insect biomass, and thereby has the potential to increase the survival of birds that use these areas for breeding, overwintering, or refueling during migration [9, 18, 30, 31]. The productivity of neotropical migrants breeding in the Pacific northwest of North America was higher in El Niño years (−ve SOI values), which are associated with wetter springtime weather along the Pacific slope from southern California to central Mexico [31, 62]. Contrary to those studies, we found little to suggest that ENSO or precipitation levels contributed to the the annual survival of chats. Similarly, other studies also found no evidence for ENSO or rainfall effects on the survival of American redstarts (*Setophaga ruticilla* [63]) and yellow warblers [7] (but see LaManna et al. [9]). An explanation for this lack of evidence could be that in years where primary productivity was poor along the migratory route, chats made more punctuated migratory bouts, enabling them to require lower fat store accumulation at stopover sites, as opposed to opting for the long-jump strategy which would demand greater metabolic expense. On its wintering ground, sites with declining Enhanced Vegetation Index (a proxy for plant productivity) had reportedly a positive impact on the within-winter survival of chats [64]. This suggests that chats have adapted well to wintering habitat types characterized by relatively low plant productivity (e.g. lowland shrub-steppe and scrub habitat), and that while strong ENSO phases and greater precipitation may promote vegetation growth in these habitats, the cascading impact on chat survival is not significant.

## Conclusions

Climate change forecasts indicate an increase in the frequency and intensity of extreme weather, including storm events, droughts, and prolonged precipitation [65]. With stronger climate fluctuations and anomalities against a backdrop of anthropogenic changes to the landscape, these stressors present a challenge for long-distant neotropical migrants during all stages of their annual cycle [8, 10, 15–18]. Our study showed that storm events were more frequent during weaker ENSO events (i.e. higher SOI values), and that westerly wind speed within the western flyway was also positively correlated with SOI values [7]. However, the direction and degree to which ENSO is affected by climate change are unclear [66]. We found that the frequency of storm events was negatively associated with chat survival, whereas GDD had a positive association. Both of those climate indices are expected to increase with climate change; whether the effects of one factor would offset the effects of another is unknown. Therefore, given such uncertainties, we are currently unable to predict with confidence the mechanistic effects of climate change on the population dynamics of chats. The interplay between these climate factors within the context of climate change will need to be further explored to elucidate how neotropical migrant bird survival will respond to a changing climate. We also recommend conducting a similar study for multiple chat populations across its range, allowing for a more complete and broader picture of the population dynamics with respect to climate conditions. Furthermore, understanding the cumulative effects of climate change and other anthropogenic threats such as habitat loss and man-made migration hazards will be critical to prioritize appropriate strategies for chats and other neotropical migratory passerines.

## Additional file

**Additional file 1.** Additional tables.
