# Supplementary material for: Wind conditions on migration influence the annual survival of a neotropical migrant, the western yellow-breasted chat (Icteria virens auricollis)
Source: BMC Ecol. 2017 Aug 10;17:29. doi: 10.1186/s12898-017-0139-7 (PMC5553749; doi:10.1186/s12898-017-0139-7)
Supplement: Supplementary file 1 — Additional file 1. Additional tables. [file 12898_2017_139_MOESM1_ESM.docx]

**Table S1** – Estimated annual survival from the simple temporal model, with standard error, lower and upper estimates.

| **Year** | **Estimate** | **Std Error** | **Lower** | **Upper** |
| --- | --- | --- | --- | --- |
| 2001-2002 | 0.405 | 0.243 | 0.087 | 0.831 |
| 2002-2003 | 0.541 | 0.181 | 0.220 | 0.831 |
| 2003-2004 | 0.693 | 0.158 | 0.345 | 0.907 |
| 2004-2005 | 0.660 | 0.151 | 0.342 | 0.879 |
| 2005-2006 | 0.600 | 0.118 | 0.364 | 0.797 |
| 2006-2007 | 0.710 | 0.129 | 0.417 | 0.893 |
| 2007-2008 | 0.488 | 0.126 | 0.262 | 0.719 |
| 2008-2009 | 0.920 | 0.128 | 0.277 | 0.997 |
| 2009-2010 | 0.420 | 0.100 | 0.245 | 0.618 |
| 2010-2011 | 0.434 | 0.116 | 0.233 | 0.658 |
| 2011-2012 | 0.427 | 0.112 | 0.233 | 0.647 |
| 2012-2013 | 0.585 | 0.154 | 0.290 | 0.830 |
| 2013-2014 | 0.378 | 0.116 | 0.188 | 0.616 |
| 2014-2015 | 0.749 | 0.153 | 0.377 | 0.936 |

**Table S2** – Correlation matrix showing the relationship between SOI and the 10 standardized climate variables. Values above and below the dashed line are Pearson’s r and the associated p-values, respectively.

|  | SOI  _MAY-APR_ | GDD _JAN-MAY_ | Breeding Ppt _OCT-APR_ | Breeding Ppt _MAY-JUL_ | Winter  Ppt _MAY-NOV_ | Winter Ppt _DEC-APR_ | U-wind _APR-MAY_ | V-wind _APR-MAY_ | Storm  _APR-MAY_ | Migration  Ppt _NOV-MAY(ARID)_ | Migration  Ppt _NOV-MAY(DESERT)_ |
| --- | --- | --- | --- | --- | --- | --- | --- | --- | --- | --- | --- |
| SOI_MAY-APR_ | --- | -0.127 | 0.148 | 0.432 | -0.155 | -0.177 | 0.605 | -0.219 | 0.522 | -0.283 | -0.16 |
| GDD_JAN-MAY_ | NS | --- | -0.232 | 0.116 | -.171 | -0.158 | -0.343 | 0.102 | -0.248 | 0.144 | 0.151 |
| Breeding  Ppt _OCT-APR_ | NS | NS | --- | 0.108 | 0.018 | 0.232 | 0.157 | 0.221 | 0.139 | -0.017 | -0.191 |
| Breeding  Ppt _MAY-JUL_ | NS | NS | NS | --- | -0.149 | -0.275 | 0.505 | 0.091 | 0.615 | -0.255 | -0.073 |
| Winter  Ppt _MAY-NOV_ | NS | NS | NS | NS | --- | 0.816 | -0.357 | -0.491 | -0.214 | -0.367 | -0.446 |
| Winter  Ppt _DEC-APR_ | NS | NS | NS | NS | 0.004 | --- | -0.210 | -0.496 | -0.168 | -0.225 | -0.393 |
| U-wind _APR-MAY_ | 0.02 | NS | NS | 0.07 | NS | NS | --- | -0.028 | 0.841 | -0.258 | -0.041 |
| V-wind _APR-MAY_ | NS | NS | NS | NS | 0.07 | 0.07 | NS | --- | -0.456 | -0.429 | -0.456 |
| Storm _APR-MAY_ | 0.06 | NS | NS | 0.02 | NS | NS | 0.002 | NS | --- | -0.213 | 0.028 |
| Migration  Ppt _NOV-MAY(ARID)_ | NS | NS | NS | NS | NS | NS | NS | NS | NS | --- | 0.927 |
| Migration  Ppt _NOV-MAY(DESERT)_ | NS | NS | NS | NS | NS | NS | NS | NS | NS | <0.001 | --- |

SOI = Southern Oscillation Index

GDD = Growing Degree Days

Ppt = precipitation

**Table S3 –** Beta estimates, standard errors and 95% confidence intervals for logit link function parameters in climate and null models in Table 4 that describe annual apparent survival and resighting probability in western yellow-breasted chats. Ppt_MAY-JUL_ is breeding ground precipitaton May - July. Ppt_DEC-APR_ is wintering ground precipitation December - April. Other parameters are as in Tables 1 - 4.

| Model | Parameter | Beta | SE | 95% CI |
| --- | --- | --- | --- | --- |
| Phi (U-wind_APR-MAY_) p(g) | Phi Intercept | 0.335 | 0.128 | 0.084, 0.586 |
|  | U-wind_APR-MAY_ | -0.356 | 0.130 | -0.611, -0.102 |
|  | p Intercept | 0.388 | 0.201 | -0.005, 0.782 |
|  | female | -1.773 | 0.329 | -2.147, -1.129 |
| Phi (GDD_JAN-MAY_ + Ppt_MAY-JUL_) p(g) | Phi Intercept | 0.364 | 0.134 | 0.102, 0.626 |
|  | GDD_JAN-MAY_ | 0.303 | 0.125 | 0.058, 0.549 |
|  | Ppt_MAY-JUL_ | -0.229 | 0.156 | -0.534, 0.076 |
|  | p Intecept | 0.395 | 0.202 | -0.001, 0.792 |
|  | female | -1.783 | 0.329 | -2.428, -1.138 |
| Phi (.) p (g) | Phi Intercept | 0.285 | 0.120 | 0.050, 0.521 |
|  | p Intercept | 0.432 | 0.202 | 0.036, 0.828 |
|  | female | -1.774 | 0.336 | -2.433, -1.115 |
| Phi (SOI_MAY-APR_) p(g) | Phi Intercept | 0.395 | 0.152 | 0.098, 0.693 |
|  | SOI_MAY-APR_ | -0.278 | 0.205 | -0.679, 0.123 |
|  | p Intercept | 0.404 | 0.203 | 0.007, 0.802 |
|  | female | -1.774 | 0.332 | -2.425, -1.123 |
| Phi (Ppt_DEC-APR_) p(g) | Phi Intercept | 0.278 | 0.119 | 0.044, 0.512 |
|  | Ppt_DEC-APR_ | -0.160 | 0.124 | -0.404, 0.083 |
|  | p Intercept | 0.456 | 0.203 | 0.057, 0.854 |
|  | female | -1.769 | 0.340 | -2.436, -1.103 |
